# COVID-19 Knowledge, Attitudes, and Practices and Perceived Risk: Cross-Sectional Mixed Methods Study

**DOI:** 10.2196/78563

**Published:** 2026-05-19

**Authors:** Aliza Bitton Ben-Zacharia, Moshe Ben-Zacharia, Jennifer Smrtka

**Affiliations:** 1 Department of Nursing, Hunter College Bellevue School of Nursing, 60 West Street, New York, NY, 10006, United States, 646-226-2616; 2 Department of Virtual Reality, The Body VR LLC, New York, NY, 10035, United States

**Keywords:** COVID-19, knowledge, attitudes, practices, percieved risk

## Abstract

**Background:**

The COVID-19 pandemic was marked by rapidly evolving and inconsistent public health messaging, contributing to confusion regarding recommended preventive behaviors. Knowledge, attitudes, and practices (KAP) and perceived risk frameworks offer a structured approach to examine how education, personal beliefs, and contextual factors influence health behaviors during public health emergencies. Vulnerable populations, such as patients with multiple sclerosis (MS), experience heightened risk perception compared with the general population, which may further shape behavioral responses.

**Objective:**

This study aimed to examine COVID-19–related KAP and perceived risk among patients with MS, health care providers, and laypeople during the first 6 months of the pandemic. The aim of mixed methods was to explore quantitative factors associated with KAP and perceived risk and to qualitatively describe participants’ perceptions and emotional responses to the pandemic.

**Methods:**

A descriptive, cross-sectional, partially mixed methods explanatory sequential design was used. Participants were recruited using convenience sampling and completed an online demographic questionnaire and a COVID-19 KAP instrument that included perceived risk items. Quantitative data were analyzed using descriptive statistics and inferential analyses to examine group differences and associations between perceived risk and preventive behaviors. Chi-square testing was applied to compare perceived risk across groups, and correlational analyses were used to examine the relationships between perceived risk and behavioral practices. Qualitative comments provided by participants were analyzed using thematic analysis to further contextualize quantitative findings and to explore perceived risk experiences.

**Results:**

A total of 148 participants were included, comprising 43 (29%) individuals with MS, 50 (33.8%) health care providers, and 55 (37.2%) laypeople. Overall, 90% (n=133) of participants demonstrated basic knowledge of COVID-19 transmission and prevention. Attitudes toward public health guidance and self-reported preventive behaviors varied across groups. Lay participants most frequently reported a moderate perceived risk of COVID-19 infection, whereas participants with MS and health care providers more commonly reported high perceived risk (*χ*²_6_=12.65, *P*=.049). Neither immunosuppressive treatment status nor vaccine hesitancy significantly predicted perceived risk. However, higher perceived risk was significantly associated with greater avoidance of crowded and public places. Qualitative analysis yielded 5 interrelated themes describing participants’ perceived risk experiences: uncertainty related to evolving scientific information; anxiety regarding personal and family safety; fear of infection and long-term consequences; vulnerability, particularly among individuals with chronic illness and frontline exposure; and accountability toward protecting others through adherence to preventive measures. These themes provided contextual insight into the emotional and cognitive processes underlying reported attitudes and behaviors.

**Conclusions:**

Knowledge of COVID-19 is associated with favorable attitudes and engagement in preventive practices across populations. Differences in perceived risk highlight the importance of tailoring risk communication and educational strategies to specific populations. KAP-focused educational interventions that explicitly address uncertainty, emotional responses, and behavioral translation may strengthen preparedness and promote sustained protective behaviors during future public health emergencies.

## Introduction

COVID-19, caused by the spread of coronavirus through respiratory droplets [1], has evolved into a pandemic, leading to rapidly changing public health messages and precautions. This has resulted in confusion among patients with chronic illnesses, health care professionals, and the public. Multiple sclerosis (MS), an autoimmune disease affecting the central nervous system, may worsen with COVID-19 [2-7], as patients with MS are more vulnerable to infection due to their immunosuppressive or immunomodulatory treatments [2-7].

Recent evidence highlights that knowledge, attitudes, and practices (KAP) during the COVID-19 pandemic critically influenced adherence to preventive behaviors and public health recommendations. Across diverse populations, adequate knowledge of COVID-19 transmission and prevention has been consistently associated with more positive attitudes and greater compliance with protective measures such as mask use, hand hygiene, and social distancing [8-10]. However, gaps between knowledge and practice remain prevalent, particularly in dynamic public health contexts. For example, cross-sectional studies among health care workers, university students, and community populations report high awareness levels—2318 (91.2%) [11] of participants recognized COVID-19 as an acute viral infection, and 2540 (99.95%) [15] correctly identified wearing masks as protective—yet adherence to recommended practices varied considerably [11-15]. Systematic reviews indicated that demographic factors, including age, education, occupation, urban residence, and access to reliable health information, significantly shape KAP outcomes. Bekele et al [16] in a systematic review of multiple cross sectional studies, reported that knowledge levels ranged from 40% to 99.5%, positive attitudes from 70% to 97.1%, and wide variation in practices, with determinants including education, occupation, income, gender, age, residence, work experience, media access, and marital status. Similarly, Rehman et al [17] found that 250 (65%) of comorbid patients demonstrated good knowledge, 219 (57%) had positive attitudes, and 196 (51%) adhered to recommended practices, with factors such as educational status (odds ratio [OR] 3.2, 95% CI 2.79‐3.58), occupation (OR 3.65, 95% CI 3.31‐4.25), urban residence (OR 2.43, 95% CI 1.65‐3.02), financial status (OR 4.02, 95% CI 3.66‐4.38), and information sources (OR 2.64, 95% CI 2.19‐3.26) significantly influencing outcomes.

These findings underscore the persistent variability in translating knowledge into preventive actions, highlighting the need for tailored public health strategies. Even in populations with high awareness and positive attitudes, practice gaps remain, emphasizing that KAP is not uniform across demographic and socioeconomic groups. Quantitative evidence demonstrates that interventions targeting education, information accessibility, and context-specific risk communication are essential to strengthen compliance with preventive measures. Moreover, continuous monitoring of KAP across different population subgroups and epidemic stages is critical for refining public health education, particularly for vulnerable groups such as patients with comorbidities, where tailored messaging can improve preventive behaviors and reduce COVID-19–related morbidity [11,16,17].

Studies on MS have shown variable results. The risk of COVID-19 in patients with MS was influenced by their knowledge, attitudes, and behaviors. Studies show that most of the patients with MS are well-informed and have good attitudes toward COVID-19 prevention, but health education programs remain essential [18]. Sahraian et al [19] found that 210 (90%) of patients with MS understood the importance of gloves and masks, though only 170 (73%) fully followed quarantine rules. Some patients still attended social events (n=40, 17%) and visited crowded places (n=65, 28%) [19]. Both laypeople and health care providers worldwide had a good understanding of COVID-19, with awareness among US public largely driven by Centers for Disease Control and Prevention (CDC) information through social media.

Most studies addressed 1 group of people, health care professionals [20-24], the public or laypeople [25-32], or patients with chronic illness [1,33]. Our study is unique as it compared the KAP of the 3 groups, health care professionals, laypeople or the public, and patients with chronic illness, which may contribute to the educational, attitudinal, and behavioral needs of each group. The aim of this study was to examine COVID-19–related KAP among health care professionals, laypeople, and individuals with MS during the first 6 months of the pandemic. Specifically, this study sought to (1) compare perceived risk of COVID-19 across these 3 groups and (2) explore qualitative themes reflecting participants’ perceptions and emotional responses to COVID-19 risk. By examining these groups concurrently, this study aimed to provide insights that may inform targeted education and risk communication strategies for diverse populations during future public health emergencies.

## Methods

### Design

This is a descriptive, cross-sectional, partially mixed methods explanatory sequential study, following the Good Reporting of a Mixed Methods Study (GRAMMS) guideline. This study concentrated on the KAP of patients with MS, health care professionals, and laypeople. The aim was to explain the quantitative survey results with qualitative themes in an explanatory sequential design. The goal of the quantitative phase was to examine the COVID-19–related KAP among health care professionals, laypeople, and patients with MS during the first 6 months of the pandemic and to compare the perceived risk of coronavirus infection across these 3 groups. The goal of the qualitative phase of this study was to identify and analyze the emergent themes reflecting participants’ perceptions of their risk related to COVID-19. The goal of the mixed methods phase was to generate integrated support for the participants’ KAP and perceived risk based on quantitative and qualitative evidence.

### Ethical Considerations

Ethical approval for this study was obtained from the institutional review board (IRB) of the City University of New York, Hunter College, Human Research Protection Program (IRB File 2020‐0299). A research consent information form was attached to the online surveys. It described the voluntary nature of the survey and participants’ right to withdraw at any time and omit answers to any of the questions. Participation after reading the information sheet constituted evidence of consent. IRB exemption was provided for this study of 148 participants aged 18 years and older, including 118 (79.7%) female participants and 27 (18.2%) male participants. No compensation was provided to participants. We have adhered to local, national, regional, and international law and regulations regarding protection of personal information, privacy, and human rights. This study was approved by the University Integrated IRB and registered at ClinicalTrials.gov (identifier: NCT07021716).

### Theoretical Framework

The knowledge-attitude-behavior (KAB) model considers that knowledge and information gained are fundamental prerequisite for behavioral changes. People are able to gain knowledge and practical skills through education. Accordingly, professionals, laypeople, and patients need to learn and get basic and advanced knowledge that can lead to the growth of positive beliefs and attitudes fortified with the acceptance of healthy behaviors [34,35]. Furthermore, the KAB framework highlights the relationship between knowledge, attitudes, and behaviors and their influence on actions [36].

### Instruments

The KAP instrument consisted of a demographic form and a KAP questionnaire. The prequestionnaire solicited basic demographic information, such as the participant’s age, gender, education level, and patients’ disease-related information. The KAP instrument was adapted from the CDC [37,38] and a previously validated Ebola KAP survey under CDC guidance with permission. Ebola-specific items were removed, and the remaining items were modified to reflect COVID-19 epidemiology, transmission routes, and recommended preventive behaviors during the early phase of the pandemic. The core KAP domains (KAP and perceived risk) were retained, and the adapted instrument was reviewed for content relevance prior to administration. The KAP survey instrument has been used nationally and internationally to explore people’s health behaviors and their lifestyle changes [26,30,32,38,39]. The survey included 50 close- and open-ended questions that took 20 to 25 minutes to complete. It covered demographics (10 items), COVID-19 knowledge (15 items), attitudes (17 items), and practices (8 items). True or false questions had “I don’t know” and “declined to answer” options, with responses categorized as correct or incorrect [40,41]. Open-ended questions were analyzed to assess the participants’ perceptions qualitatively.

Perceived risk for contracting COVID-19 was assessed using a 5-point scale (0=no risk at all, 1=small risk, 2=moderate risk, 3=high risk, and 4=don’t know). Perceived risk of COVID-19 was assessed as a categorical variable. It was analyzed as dichotomous variable (low risk vs moderate-t0-high risk) and as 4-categorical variable (low risk, moderate risk, high risk, and don’t know). Perceived risk was assessed and analyzed in each group, comprising patients with MS, health care professionals, and laypeople.

### Data Collection

The population for this study included health care professionals (nurses and physicians), patients with MS, and laypeople from the northeast region in the United States. It included participants from New York, Connecticut, and Massachusetts. A convenience sampling approach was used to enhance recruitment. The participants were recruited using blast emails through health centers, MS centers, and community resources. Data were collected via an online self-reported questionnaire from April 2020 to September 2020. The surveys were completed online using anonymous and confidential software, that is, a “Monkey” survey. The inclusion criteria of the study included adult people (≥18 y), consisting of patients with MS, health care professionals (nurses and physicians), and adult laypeople. The study did not include any exclusion criteria.

### Data Analysis

Descriptive analyses were used to characterize the sample. Chi-square and Kruskal-Wallis tests assessed associations between demographics, KAP, and perceived risk. Kruskal-Wallis test compared KAP across patients with MS, health care professionals, and laypeople. Logistic regression assessed the perceived risk as a dependent dichotomized variable (low and moderate-to-high risk) and immunocompromised status or vaccination hesitancy as independent variables when controlling for age, sex, and education. Statistical significance was set at *P*<.05.

A qualitative thematic analysis was performed and coded on participants’ comments by 2 researchers (AB-Z and JS) to identify key themes reaching data saturation. Participants’ open-ended comments were analyzed using thematic analysis following the framework described by Braun and Clarke [42-45], with consideration of their updated recommendations for good practice in thematic analysis. The analysis followed the 6-phase approach, including familiarization with the data, generation of initial codes, identification of candidate themes, review and refinement of themes, definition and naming of themes, and final reporting. Two researchers independently reviewed and coded participants’ written responses to ensure analytic rigor. An inductive approach was used, allowing themes to emerge directly from the data rather than applying predetermined categories. Coding discrepancies were discussed and resolved through consensus to refine the coding framework and ensure consistent interpretation of the data. To enhance the trustworthiness of the qualitative analysis, several strategies were applied. Credibility was supported through independent coding and collaborative discussions between researchers during the analytic process. Dependability was strengthened through systematic documentation of coding decisions and theme development. Confirmability was addressed by grounding interpretations in participants’ responses and maintaining transparency in the analytic procedures. Transferability was supported by providing contextual information about the study population and setting to allow readers to assess the potential applicability of the findings to other contexts. Data saturation was considered achieved when no additional themes or meaningful insights emerged from the participants’ responses. Reflexivity was also considered throughout the analysis, with the researchers acknowledging their professional backgrounds and engaging in regular discussions to minimize potential bias and ensure that interpretations remained closely aligned with participants’ perspectives [42-45].

## Results

### Quantitative Analysis

The study included 148 participants, 43 (29%) patients with MS, 50 (33.8%) health care professionals (90% nurses and 10% physicians), and 55 (37.2%) laypeople (Tables1  2). The majority of the participants were female and had college education. The characteristics of the sample and demographics characteristics of the participants are given in Tables1  2, respectively. Participant responses revealed a high level of awareness and knowledge regarding the COVID-19 pandemic, including its etiology, transmission, and clinical manifestations (Table 3). For example, 116 (91.3%) participants correctly identified the viral pathogen responsible for COVID-19 and 121 (94.5%) knew its transmission (Questions 18 and 19; Table 3). Despite their high basic knowledge, 53.4% (n=79) of respondents indicated a need for further education concerning medical care and available treatment options for COVID-19, underscoring persistent knowledge gaps that can influence decision-making and health care—seeking behaviors (Tables1^3^).

**Table 1.** Characteristics of the sample (N=148).

Characteristic	Value
Participants, n (%)	
Laypeople	55 (37.2)
Health care professionals	50 (33.8)
Patients with MS^a^	43 (29)
Age (y) (continuous), mean (SD)	49.5 (15.3)
Age group (y), n (%)	
18‐30	18 (12.3)
31‐45	36 (24.7)
46‐60	56 (37.3)
>60	36 (24.7)
Sex	
Female	118 (79.7)
Male	27 (18.2)
Other	2 (1.4)
Level of education, n (%)	
High school degree	9 (6.5)
Bachelor’s degree	55 (37.2)
Master’s degree	55 (37.2)
Doctoral degree	13 (8.8)
Other	15 (10.1)
Religion, n (%)	
Catholic	54 (36.5)
Jewish	39 (26.5)
Buddhist	4 (2.8)
Nonreligious	38 (25.5)
Other	12 (8.4)
Heard about COVID-19 infection, n (%)	
Yes	137 (92.6)
No	10 (6.8)
Tested positive for COVID-19, n (%)	
Yes	8 (5.4)
No	140 (94.6)
Are you immunocompromised, n (%)	
Yes	33 (22.3)
No	69 (46.6)
Not applicable	29 (19.6)
I don’t know	9 (6.1)
COVID-19 information sources, n (%)	
Relatives and friends	82 (55.4)
Radio	75 (50.7)
Television	126 (85.1)
Health care professionals	133 (89.9)
Social media	39 (26.4)
Governmental officials	56 (37.8)
Newspapers and flyers	93 (62.8)
Internet and browsers	126 (85.1)
Mobile phone and texts	40 (27)
Areas where more COVID-19 information is needed, n (%)	
Cause and origin of the disease	30 (20.3)
Signs and symptoms of the disease	30 (20.3)
Ways to prevent the disease	49 (33.1)
Medical care and treatment options	79 (53.4)

a MS: multiple sclerosis.

**Table 2.** Demographic characteristics of participants by group (N=148).^a^

Characteristic	Patients with MS^b^ (n=43)	Laypeople (n=55)	Health care professionals (n=50)	Missing data
Age (y), mean (SD)	54.10 (12.37)	52.15 (17.25)	43.76 (13.36)	1
Age group (y), n (%)				5
18‐30	1 (2.5)	6 (11.5)	9 (17.6)	
31‐45	5 (12.5)	12 (23.1)	19 (37.3)	
46‐60	24 (60)	16 (30.8)	16 (31.4)	
>60	10 (25)	18 (34.6)	7 (13.7)	
Sex, n (%)				4
Female	33 (80.5)	38 (71.7)	45 (90)	
Male	8 (19.5)	14 (26.4)	4 (8)	
Other	0 (0)	1 (1.9)	1 (2)	
Education level, n (%)				3
High school diploma	7 (17.1)	1 (1.9)	0 (0)	
Bachelor’s degree	15 (36.6)	23 (43.4)	17 (33.3)	
Master’s degree	9 (22)	20 (37.7)	26 (51)	
Doctoral degree	3 (7.3)	2 (3.8)	7 (13.7)	
Other	7 (17.1)	7 (13)	1 (2)	
Religion, n (%)				3
Catholic	18 (43.9)	17 (32.1)	19 (37.2)	
Jewish	10 (24.4)	21 (39.6)	6 (11.8)	
Buddhist	1 (2.4)	1 (1.9)	2 (3.9)	
Nonreligious	9 (22)	10 (18.8)	19 (37.3)	
Other	3 (7.3)	4 (7.6)	5 (9.8)	
Heard of COVID-19, n (%)				3
Yes	39 (95.1)	49 (92.5)	47 (92.2)	
No	2 (4.9)	4 (7.5)	4 (7.8)	
Tested positive for COVID-19, n (%)				2
Yes	1 (2.3)	3 (5.5)	4 (8)	
No	40 (93)	52 (94.5)	46 (92)	
Immunocompromised status, n (%)				8
Yes	26 (63.4)	3 (6.1)	4 (8)	
No	7 (17.1)	33 (67.3)	29 (58)	
Not applicable	1 (2.4)	12 (24.5)	16 (32)	
I don’t know	7 (17.1)	1 (2)	1 (2)	

a Percentages may not total 100 due to rounding.

b MS: multiple sclerosis.

**Table 3.** Knowledge, attitudes, and practices differences among the 3 groups of participants.

Knowledge, attitudes, and practices^a^	Full sample, n (%)	Laypeople, n (%)	Patients with MS^b^, n (%)	Health care professionals, n (%)	Effect size Cramer *V*
Knowledge					
12. COVID-19 exists globally and locally	147 (99.3)	53 (36.6)	41 (28.3)	51 (35.2)	Constant
13. Possible to recover and survive	126 (99.2)	46 (36.5)	38 (29.2)	43 (34.1)	0.136
14. Possible to have COVID-19 without issues	128 (100)	46 (35.7)	38 (29.7)	44 (34.4)	Constant
18. The cause for the COVID-19—virus	116 (91.3)	40 (34.5)	35 (30.2)	41 (35.3)	0.117
19. How does a person get COVID-19—droplets of coughing and sneezing	121 (94.5)	43 (35.5)	36 (29.8)	42 (34.7)	0.072
20. What is the worse sign and symptom of COVID—breathing difficulty	125 (97.7)	46 (36.8)	36 (28.8)	43 (34.4)	0.162
21. Preventing COVID-19 with blood and body precautions	44 (29.7)	28 (35)	12 (31.6)	28 (35)	0.048
22. Preventing COVID-19 by avoiding crowds	104 (81.3)	36 (34.7)	35 (33.7)	33 (31.7)	0.180
23. Protection by not touching other	117 (89.9)	43 (36.8)	35 (29.9)	39 (33.3)	0.097
24. COVID-19 can be better treated in a facility	67 (45.3)	19 (28.4)	22 (32.8)	26 (38.8)	0.169
25. Better to go to a facility since family is protected	59 (39.9)	20 (33.9)	17 (28.8)	22 (37.3)	0.127
26. COVID-19 prevention by bathing with hot water	121 (83.1)	43 (35.5)	32 (26.4)	46 (38)	0.177
27. COVID-19 is transmitted via air	116 (79.7)	44 (37.9)	31 (26.7)	41 (35.3)	0.106
28. COVID-19 can be transmitted by mosquito bites	93 (72.7)	31 (33.3)	27 (29)	35 (37.6)	0.196
29. Complementary medicine can treat COVID-19	76 (52.7)	22 (28.9)	23 (30.3)	31 (40.8)	0.198^c^
30. Spirituality and religion can treat COVID	113 (88.3)	39 (34.5)	34 (30.1)	40 (35.4)	0.081
Attitudes					
31. What happens if COVID suspected—to the hospital	126 (85.1)	39 (30.1)	43 (34.9)	44 (34.9)	0.196
32. COVID-19 diagnosis must be admitted	105 (71.6)	38 (36.2)	27 (25.7)	40 (38.1)	0.165^c^
33. COVID-19 exposed must be quarantined	133 (91.2)	49 (36.8)	37 (27.8)	47 (35.3)	0.086
34. Suspicion that you had COVID-19, hospitalized	53 (35.8)	18 (34)	13 (24.5)	22 (41.5)	0.273^d^
35. Fever, would you go to a facility	73 (50)	23 (31.5)	21 (28.8)	29 (39.7)	0.093
36. Family suspected, hospital or avoid contact	117 (91.4)	43 (36.8)	35 (29.9)	39 (33.3)	0.078
37. Complementary medicine cure COVID	66 (51.6)	21 (31.8)	22 (33.3)	23 (34.8)	0.187
39. A student recovered COVID-19, put others at risk	70 (48.6)	19 (27.1)	16 (22.9)	35 (50)	0.248^d^
40. A person recovered COVID-19, back in community	136 (93.2)	49 (36)	37 (27.2)	50 (36.8)	0.170^c^
41. Shop owner, recovered, shop there buying produce	88 (60.8)	32 (36.4)	22 (25)	34 (38.6)	0.116
Practices					
37. Actions to prevent COVID-19	144 (97.9)	53 (36.8)	40 (27.8)	51 (35.4)	0.133
38. Behavior to avoid infection and avoid public places	144 (97.3)	45 (36.3)	36 (29)	43 (34.7)	0.080
42. COVID-19 vaccine—accept	115 (89.8)	41 (35.7)	36 (31.3)	38 (33)	0.112
43. COVID-19 vaccine—accept for your children	83 (93.7)	31 (37.3)	24 (28.9)	28 (33.7)	0.047
44. COVID treatment only with animal research—accept	32 (25)	11 (34.4)	13 (40.6)	8 (25)	0.121

a KAP questions adapted from CDC/Ebola survey.

b MS: multiple sclerosis.

c .05<*P*<.10.

d *P*<.05.

A detailed analysis of participant responses revealed considerable variability in attitudes and behaviors related to COVID-19 infection and recovery. Specifically, a substantial proportion of respondents indicated engagement in preventative measures and a willingness to reintegrate individuals into their communities following recovery from COVID-19, demonstrating a generally accepting attitude toward postinfection social reintegration (Questions 37, 39, 40, and 41; Tables3  4). Additionally, COVID vaccination hesitancy did not predict the perceived risk of contracting the virus among the participants when controlling for age, sex, and education (*B*=−0.450, *W*[1]=0.505, *P*=.48). However, vaccination acceptance rates were notably high, as 115 (89.8%) participants expressed their willingness to receive an approved COVID-19 vaccine (Question 42; Tables3  4). Among the 95 (70%) participants identified as having children, 83 (87%) stated their intent to vaccinate their children, indicating a strong overall endorsement of vaccination as a preventive strategy (Question 43; Tables3  4). Older participants were more likely to accept an approved COVID vaccine (*χ*^2^_2_=5.43, *P*=.07).

**Table 4.** Association between knowledge, attitudes, and practices and perceived risk.

Knowledge, attitudes, and practices correct responses^a^	*χ*^2^ (*df*)	*P* value	Effect size Cramer V
Knowledge			
12. COVID-19 exists globally and locally	Constant (Yes)	—^b^	—
13. Possible to recover and survive	0.250 (1)	.62	0.043
14. Possible to have COVID-19 without signs and symptoms	Constant	—	—
21. Preventing COVID-19 with blood and body precautions (No)	3.107 (1)	.09^c^	0.151
22. Preventing COVID-19 by avoiding crowds	7.469 (2)	.02^d^	0.234
23. Protection by not touching others	1.813 (2)	.40	0.115
24. COVID-19 can be better treated in a facility (No)	9.800 (2)	.02^d^	0.267
25. Better to go to a facility since family is protected (No)	5.708 (2)	.13	0.204
26. COVID-19 prevention by bathing with hot water (No)	3.757 (2)	.15	0.166
27. COVID-19 is transmitted through air	2.357 (2)	.31	0.131
28. COVID-19 can be transmitted by mosquito bites (No)	0.002 (1)	.96	0.004
29. Complementary medicine can treat COVID-19 (No)	5.320 (2)	.15	0.197
30. Spirituality or religion can treat COVID-19 (No)	4.932 (2)	.18	0.190
Attitudes			
32. COVID-19 diagnosis must be admitted (No)	1.635 (2)	.65	0.109
33. COVID-19 exposed must be quarantined	0.832 (2)	.66	0.078
34. Suspicion that you had COVID-19, then would you get admitted (No)	7.595 (2)	.05^c^	0.235
35. Fever, would you go to a facility	4.674 (2)	.10^c^	0.185
39. A student recovered from COVID-19 put others at risk at school (No)	1.199 (2)	.88	0.094
40. Shop owner recovered from COVID-19, you will shop there buying produce	0.396 (2)	.94	0.054
41. A person that recovered COVID-19, back in community	1.540 (2)	.46	0.106
Practices			
37. Actions to prevent COVID-19	Constant (Yes)	—	—
42. COVID-19 vaccine—accept	1.313 (2)	.52	0.098
43. COVID-19 vaccine—accept for your children (n=95 children had, 70%)	7.245 (2)	.06^c^	0.230
44. COVID-19 treatment only with animal research—accept it	4.713 (2)	.19	0.185

a KAP questions adapted from CDC/Ebola survey.

b Not applicable.

c .05<*P*<.10.

d *P*<.05.

A comprehensive analysis of participant responses across the 3 study groups—laypeople, individuals diagnosed with MS, and health care professionals—revealed a few statistically significant differences in overall KAP and perceived risk related to COVID-19 (Table 4). There was a statistically significant difference between the 3 groups associated with their perceived risk (*χ*^2^_6_=12.65, *P*=.049) (Table 5). Thus, lay participants have reported mostly a moderate perceived risk for COVID as compared with patients with MS and health care providers who reported mainly a high risk to contract the virus (Tables 3-5). Additionally, health care professionals were significantly more likely than the other groups to agree that people could safely return to school or work following recovery from COVID-19 without posing a risk to others (*P*<.05). Further analysis highlighted considerable uncertainty among participants regarding the appropriate facilities for COVID-19 treatment and the safety of reintegration into community settings postrecovery (Questions 24, 25, 34, 35, 39; Tables3  4). Moreover, uncertainty was prevalent in responses related to novel treatment options, particularly those studied exclusively in animal models (Question 44; Tables3  4).

**Table 5.** Perceived risk of coronavirus/COVID-19 among the participants.

COVID-19 perceived risk	Laypeople, n (%)	Patients with multiple sclerosis, n (%)	Health care professionals, n (%)	Total, n (%)
No risk	0 (0.0)	1 (2.4)	0 (0.0)	1 (0.7)
Mild risk	7 (13.2)	12 (29.3)	7 (14.0)	26 (18.1)
Moderate risk	36 (67.9)	16 (39.0)	29 (58.0)	81 (56.3)
High risk	6 (11.3)	8 (19.5)	13 (26.0)	27 (18.8)
Unsure	4 (7.5)	4 (9.8)	1 (2.0)	9 (6.3)
Total	53 (100)	41 (100)	50 (100)	144 (100)

Perceived risk analysis among the participants have shown variable results. There was a trend toward a negative correlation between perceived level of risk and participant age, with older individuals demonstrating lower perceived susceptibility to COVID-19 infection (*t*_143_=−1.781, *P*=.06). Chi-square bivariate analysis did not show a statistically significant association between gender (female, male, nonbinary) (*χ*^2^_2_=4.452, *P*=.11), religious affiliation (*χ*^2^_1_=0.698, *P*=.40), and education (*χ*^2^_1_=0.660, *P*=.42) and perceived risk. There was no statistically significant association between professional specialty and self-reported risk level, suggesting that risk perception was consistent across various health care disciplines. Immunocompromised status did not predict perceived risk levels when controlling for age, sex, and education (*B*=−442, *W*[1]=.818, *P*=.33). Markedly, perceived risk demonstrated a statistically significant association with behavioral modifications aimed at mitigating infection risk (Table 4). Specifically, participants with moderate to high perceived susceptibility were significantly more likely to engage in risk-averse behaviors, including avoiding crowded places, a decreased likelihood of seeking care at a medical facility, or visiting a hospital if they suspected COVID-19 infection (Questions 22, 24, 34; Tables3  4).

### Qualitative Analysis

A qualitative thematic analysis was conducted to explore participants’ perceptions of risks associated with COVID-19, following the analytic framework described by Virginia Braun and Victoria Clarke. Participants’ open-ended responses were first read repeatedly to achieve data familiarization, after which 2 researchers (AB-Z and JS) independently generated initial codes. Through an iterative and reflexive process of reviewing, comparing, and refining codes, patterns of shared meaning across the dataset were identified and organized into candidate themes. These themes were subsequently reviewed and refined to ensure internal coherence and clear distinction between themes. The analysis revealed several key themes reflecting participants’ perceptions of pandemic-related risk, including uncertainty, anxiety, fear, vulnerability, and accountability. Thematic saturation was reached when no additional patterns or themes emerged from the data. Differences and similarities in thematic expressions were examined across the 3 participant groups: individuals with MS, health care professionals, and lay community members. Across groups, dominant themes centered on feelings of uncertainty and heightened anxiety about contracting the virus, concerns about vulnerability to infection, and a perceived sense of personal and collective responsibility for infection prevention.

Participants expressed heightened concern about their perceived risk, particularly due to residing in highly affected areas, such as New York City and surrounding regions. The density of the population and the high prevalence of COVID-19 cases in these areas amplified their perceived susceptibility:

*NYC has more cases than anywhere else in the US, and that puts everyone at risk.*

*I live in a densely populated city.*

*I think we are all at risk since thousands have contracted this virus.*

*I live in New Jersey, close to NY, which has a high rate of infection.*

Another recurring theme was the perception of vulnerability due to age. Several participants highlighted concerns about their increased susceptibility to COVID-19 due to being older adults or cohabiting with elderly family members or young children. These responses illustrated an awareness of age-related risk factors:

*I am highly concerned since I am over 60 and live near NYC.*

*I live with my elderly mother, and we are both at high risk.*

*I have young children at home, and I have to protect them from being exposed to the virus.*

Work-related exposure also emerged as a critical factor, with participants expressing concern and vulnerability over their occupational and social interactions. Essential workers and those with frequent public interactions perceived themselves at an elevated risk due to the nature of their jobs. Representative comments included:

*I have contact with numerous people at work and I have to continue going to work because I am an essential worker.*

*I am an essential worker and still mix with people from different backgrounds and locations.*

*Through personal interaction and work, I come into contact with people from different backgrounds who might be exposed.*

*As a nurse, I have experienced many events in my career, but this is beyond anything I have experienced. This pandemic makes me feel very vulnerable.*

Patients with MS reported feeling particularly vulnerable, attributing their heightened risk to both their condition and the immunosuppressive nature of their medications. Their narratives underscored concerns about compromised immunity and the necessity for extra precautions. Statements reflecting this vulnerability included:

*I’m immunocompromised and at higher risk than most people. I need to be extra cautious.*

*I am on a medication that lowers white blood cells, and it puts me at risk for infections. I have to continue the medication following my MS team.*

*My immune system is compromised due to my MS. I am concerned about any imminent infection that will affect my disease.*

*I was very concerned about contracting the virus, therefore I decided to see my providers only via telemedicine.*

Participants collectively acknowledged a shared responsibility in mitigating the spread of COVID-19. Some emphasized the importance of protective measures such as wearing masks and gloves, illustrating a sense of communal accountability:

*All people need to take some sort of responsibility, like wearing a mask and gloves, because they can spread it.*

*Everyone needs to follow precautions set by major organizations and institutions.*

*As a healthcare professional, I feel responsible of teaching others about precautions and the importance of wearing masks and other measures.*

The qualitative findings reflect a complex interplay of geographic, occupational, medical, and demographic factors shaping participants’ perceptions of risk. The overarching themes of high-risk environments, personal vulnerability, and the perceived necessity for protective behaviors provided a deeper understanding of how individuals navigated the psychological and practical challenges posed by the pandemic.

## Discussion

### Principal Results

Participants in this study exhibited a high level of knowledge regarding COVID-19, including its etiology, transmission modes, and symptomatic presentation. However, knowledge gaps persisted in areas related to appropriate health care facilities, treatment options, and safe reintegration into the community following recovery. Furthermore, analysis of participant responses revealed generally positive attitudes toward COVID-19 prevention and postinfection reintegration, with high engagement in preventative behaviors and strong acceptance of vaccination for themselves (n=115, 89.8%) and their children (n=82, 87%), (n=95, 70% had children), particularly among older participants. Differences emerged across study groups, with lay participants reporting moderate perceived risk, whereas individuals with MS and health care professionals reported higher perceived risk, and health care professionals were more confident about safe reintegration into schools or workplaces. Overall, perceived risk was not significantly associated with gender, education, religious affiliation, immunocompromised status, or health care specialty, though older participants tended to perceive lower susceptibility. Importantly, higher perceived risk consistently predicted greater adoption of risk-averse behaviors, including avoidance of crowded settings and medical facilities, while notable uncertainty remained regarding appropriate treatment facilities, postrecovery community reintegration, and novel interventions, highlighting areas for targeted education and guidance.

The qualitative findings provided further context for these quantitative patterns. Thematic analysis of open-ended responses identified several dominant themes shaping participants’ perceptions of pandemic risk, including uncertainty, anxiety, fear, vulnerability, and accountability. Taken together, the integrated findings suggest that although participants possessed substantial baseline knowledge and demonstrated strong engagement in preventive behaviors, emotional responses to the pandemic, particularly uncertainty and perceived vulnerability, played a critical role in shaping risk perception and behavioral choices. Persistent uncertainty regarding treatment pathways and postrecovery reintegration further highlights the need for clear, targeted public health communication to address these specific areas of concern.

### Comparison with Prior Work

These findings align with previous national and international studies that have documented considerable public awareness of pathophysiology and transmission dynamics of COVID-19 [1,17,23-25,35]. The sources of this knowledge likely stem from various platforms, including social media, governmental communications, and clinical medical resources. Furthermore, the World Health Organization [46,47] and CDC have emphasized the utility of KAP models serving as frameworks for public health education programs. Remarkably, the KAB model stresses the importance of knowledge as a foundation for behavioral shifts [36], and public health authorities should use creative strategies to encourage social change and improve attitudes and behaviors [1,19,33-35]. Additionally, KAPs regarding COVID-19 are interconnected, but knowledge alone does not always lead to preventive behaviors [1,19,27,33,34,48]. Accordingly, our findings contribute to the growing body of literature on COVID-19 awareness and highlight the importance of targeted educational interventions to address persistent knowledge gaps.

In our study, patients with MS and health care professionals had a high perceived risk to contract the virus, while laypeople had a moderate risk (Table 5). Research shows that a higher risk perception leads to better adherence to preventive measures [49]. Furthermore, patients with MS were highly concerned about becoming infected during the local peak of the COVID-19 pandemic [50]. Most of our study participants have favored vaccination for themselves and their children, once a vaccine was available. Research has shown that COVID-19 vaccine hesitancy decreased during the pandemic, although 1 in 5 adults with MS were hesitant in early 2021 [51]. Behaviors that deviated from originally recommended care for patients with MS were common and often self-initiated, but patients were overall compliant with continuing their disease-modifying therapies [50,51].

Qualitatively, our study has identified the themes of uncertainty, anxiety, fear, vulnerability, and accountability associated with the perceived risk of patients with MS, health care professionals, and laypeople. Thus, our study revealed several psychological themes that are prevalent in individuals’ perceptions of COVID-19 risk. These themes indicate the multifaceted emotional responses that individuals experience when confronted with the possibility of infection [50]. Uncertainty reflects the unpredictable nature of the virus as well as MS, while anxiety and fear stem from concerns about personal and family health, and the potential for severe outcomes. Contrastingly, vulnerability highlights a sense of exposure and the limited control over preventing infection in a global pandemic. Jointly, these themes [50] underscore the need for support services that address these psychological burdens, particularly in public health responses aimed at easing COVID-19–related fears and anxiety. Similarly, Cadwgan and her colleagues [52] identified a few themes to include “uncertainty and anxiety,” “exacerbation of inequalities,” and “care provision: reaction, adaptation, and innovation”. Mejdahl and colleagues [53] noted themes of vulnerability and uncertainty related to the infection risk and social distancing. Comparably to our study, other studies highlighted the uncertainty and anxiety feelings with the risk of acute illness and worsening of the participants’ chronic illnesses [52]. Our study participants, particularly the patients with MS, were concerned about their immunosuppressive status and the potential impact of COVID-19 on their MS status [50]. Other studies identified themes such as fear, stigma, and social isolation [50,53,54].

The COVID-19 pandemic has had a profound and far-reaching impact on global public health systems, revealing both strengths and vulnerabilities in health infrastructure and communication. Beyond the immediate morbidity and mortality associated with SARS-CoV-2 infection, the pandemic disrupted routine health care services, exacerbated health disparities, and strained workforce capacity [55]. Public health agencies faced unprecedented challenges in disease surveillance, contact tracing, testing, and vaccine distribution, while also contending with widespread misinformation and public skepticism. This study has underscored the vast knowledge that the participants had during the early period of the pandemic, leading to partially following precautionary measures. In addition, this study underlined the critical need for sustained investment in public health preparedness, and the development of resilient health systems capable of responding to the needs of vulnerable populations, such as patients with MS and other chronic illnesses.

### Limitations

This study has several limitations that should be considered when interpreting the findings. The cross-sectional design, which captures a single point in time, does not allow for causal conclusions. A cross-sectional design was selected to efficiently assess and compare KAP related to COVID-19 among patients with MS, health care providers, and the general public during the pandemic period. The convenience sampling used limits representativeness and may introduce researcher and selection biases. Convenience sampling was employed due to logistic constraints and restricted access to participants during the pandemic, as well as the limited and specialized clinical population of individuals with MS, which made probability-based sampling difficult to implement within the study time frame. Additionally, the sample size within each group was relatively modest (n=~50), which may limit the generalizability of the results to broader populations. In particular, individuals with MS represent a specialized clinical population, and recruitment was limited to a defined sampling frame during the COVID-19 pandemic, when institutional restrictions and increased clinical demands have affected participant availability. Nevertheless, equal group sizes were intentionally used to facilitate balanced comparisons among patients, health care providers, and lay participants, thereby improving the internal validity of between-group analyses. While the quantitative methods provided valuable data on trends and patterns during the pandemic, integrating qualitative methods enriched the understanding by providing context and depth to the results including thematic saturation. Additionally, the study was conducted in New York, Connecticut, and Massachusetts, primarily with highly educated participants, so that the findings may not apply to the broader population. Lastly, demographic data on race and ethnicity and economic measures were not collected, limiting insight into underserved populations.

### Conclusions

The findings of this study underscore the critical role of KAP in shaping behaviors that reduce the spread of COVID-19 and other infectious diseases. While participants generally demonstrated good understanding and positive attitudes, variability in adherence to preventive measures highlights the need for tailored interventions. Perceived risk influenced protective behaviors, suggesting that public health messaging must address both awareness and personal relevance to effectively motivate compliance. Vulnerable populations, such as patients with MS, may benefit from targeted education and support, while health care professionals can leverage their trusted position to guide safe practices within communities. Understanding the KAP across different groups allows public health authorities to design nuanced strategies that enhance preparedness, encourage sustained behavior change, and strengthen resilience against future pandemics.

## Abbreviations

CDC: Centers for Disease Control and Prevention
GRAMMS: Good Reporting of a Mixed Methods Study
IRB: institutional review board
KAB: knowledge-attitude-behavior
KAP: knowledge, attitudes, and practices
MS: multiple sclerosis
OR: odds ratio
